# Cis-abienol from tobacco trichomes to ambergris-like compounds: advances in biosynthesis, fermentation, and industrial applications

**DOI:** 10.3389/fbioe.2025.1713206

**Published:** 2026-01-09

**Authors:** Wenting Wang, Xinlong Zhang, Wei Hu, Shen Huang, Robina Manzoor, Aamir Rasool

**Affiliations:** 1 Wuhan Vocational College of Software and Engineering (Wuhan Open University), Wuhan, China; 2 School of Tobacco Science and Engineering, Zhengzhou University of Light Industry, Zhengzhou, China; 3 Department of Biotechnology and Bioinformatics, Lasbella University of Agriculture, Water and Marine Science, Uthal, Pakistan; 4 Institute of Biochemistry, University of Balochistan, Quetta, Pakistan; 5 Jamil-ur-Rahman Center for Genome Research, Dr. Panjwani Center for Molecular Medicine and Drug Research, International Center for Chemical and Biological Sciences, University of Karachi, Karachi, Pakistan

**Keywords:** ambergris, bioconversion, cis-abienol, metabolic engineering, tobacco trichomes

## Abstract

Cis-abienol is found in small quantities in plants like *Nicotiana tabacum* and *Abies balsamea*. It serves as a precursor for the synthesis of ambergris-like compounds, including ambroxide and ambreinolide, which are highly valued in the perfume industry for their long-lasting fixative properties and distinctive scent profile. This review summarises current progress in understanding (i) the biosynthetic pathways, chemical properties, and microbial or enzymatic degradation of cis-abienol in tobacco, particularly its production in glandular trichomes and its degradation during curing; and (ii) the chemical conversion of tobacco-derived cis-abienol and its analogues, such as sclareol, into ambrox, ambreinolide, and related fragrance compounds through oxidation, reduction, and cyclisation reactions. The bioconversion of cis-abienol or sclareol into ambergris-like compounds (AmbLCs) represents a sustainable and environmentally friendly alternative to traditional chemical methods; however, efficient biotechnological approaches for the direct biodegradation of cis-abienol into ambergris analogues remain underdeveloped. Future work should focus on metabolic engineering, enzyme discovery, and microbial pathway optimization to enhance the efficiency of these transformations, thereby laying the foundation for utilizing tobacco as a sustainable source of AmbLCs.

## 1 Introduction

**FIGURE 1:**
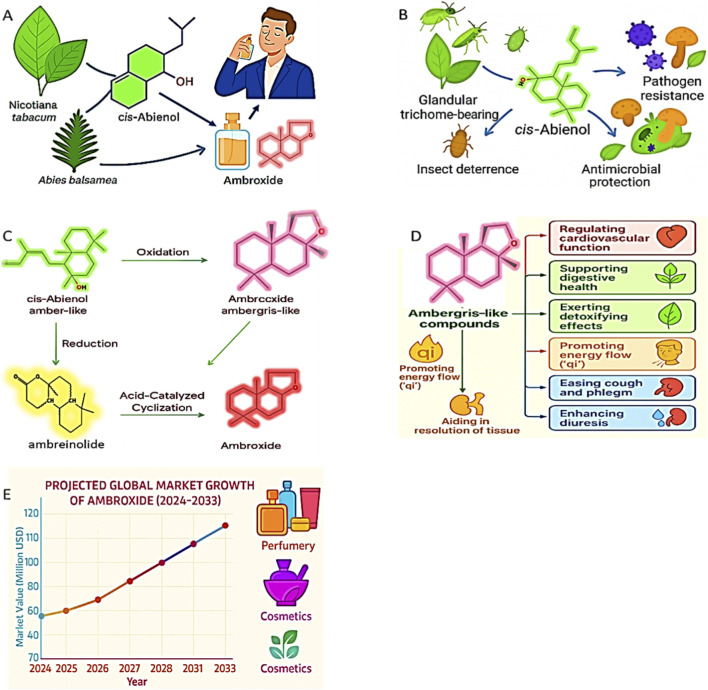
**(A–E)** Multifunctional role of cis-abienol in plants: biosynthesis, defence, and application: **(A)** Biosynthetic origin of cis-abienol from *N. tabacum* and *A. balsamea*, its conversion into ambroxide, and final use in perfumery. **(B)** Defensive roles of cis-abienol in glandular trichome-bearing plants, contributing to insect deterrence, antimicrobial protection, and resistance against a range of pathogens. **(C)** Presents the chemical conversion pathway of *cis*-Abienol to Ambroxide via oxidation, reduction, and acid-catalysed cyclisation. **(D)** A flow diagram showing the traditional medicinal functions of AmbLCs derived from *cis*-Abienol. Key effects include detoxification, anti-inflammatory action, and enhanced fragrance fixative properties. **(E)** Projects the global market growth of Ambroxide from 2024 to 2033, showing rising demand in perfumery and cosmetics.

Cis-abienol is a labdane-type diterpenoid (labTD) alcohol secreted on the surfaces of plants like *Nicotiana tabacum* and *Abies balsamea* (Wu et al., 2019; Zerbe et al., 2012a). Cis-abienol plays a key role in the fragrance profile of these plants and serves as a precursor to AmbLCs, including ambroxide and ambreinolide (Wu et al., 2019; Zerbe et al., 2012a). Gray et al. first isolated cis-abienol from the oleoresin of *Abies balsamea* in 1964 and confirmed its chemical structure as a bicyclic tertiary diterpene alcohol with a conjugated double bond (Gray and Mills, 1964; Zerbe et al., 2012a) (Figure 1A). Trans-abienol reported in GC-MS analyses seems to result from the thermal isomerization of cis-abienol, rather than from its natural presence (Zerbe et al., 2012a). It is also secreted in the glandular trichomes of *N. tabacum* leaves, where its accumulation contributes to both the chemical ecology and aroma characteristics of the plant (Han et al., 2025). Studies have shown that cis-abienol is a major component of the essential oils of coniferous species, particularly *A. balsamea* (balsam fir) and *A. sibirica* (Ancuceanu et al., 2023; Duquesnoy et al., 2010).

Cis-abienol plays a vital role in the chemical defense system of plants, especially in glandular trichome-bearing species and conifers (Glas et al., 2012) (Figure 1B). Cis-abienol is often produced in response to biotic stress caused by bacterial pathogens (*Ralstonia solanacearum*, *Pseudomonas syringae, Ips typographus),* fungal pathogens (*Alternaria alternata*, *Fusarium oxysporum, Rhizoctonia solani, Heterobasidion annosum, Armillaria spp., Fomes spp),* viral pathogens (tobacco mosaic virus), and insect herbivores (*Myzus persicae*, *Helicoverpa armigera*) (Huchelmann et al., 2017; Zerbe et al., 2012a). Hence, cis-abienol plays an essential role in enhancing plant survivability and ecological fitness.

Cis-abienol can be converted into AmbLCs through oxidation, reduction, and acid-catalysed cyclisation reactions (Figure 1C). These AmbLCs are also highly valued in the fragrance industry for their characteristic scent and fixative properties (He et al., 2024; Ma et al., 2024; Ncube et al., 2020). Beyond their importance as fragrance ingredients, ambergris-like compounds derived from cis-abienol also possess reported pharmacological and traditional medicinal significance, which further increases interest in their sustainable production (Ahmadi et al., 2024; Beeh et al., 2008; Cavalu et al., 2022; Fernández et al., 2024; Malerba and Ragnoli, 2008; Riddle and Steiner Verlag, 1964; Rowland et al., 2019; Russo et al., 2023; Wilde et al., 2020) (Figure 1D).

Cis-abienol is gaining industrial significance as a precursor for fragrance molecules, and its economic profile further highlights this relevance. Cis-abienol is, however, a high-cost intermediate, and quality lab-grade reagent costs over USD 4,000/g, whereas its main ambergris-like derivative, ambroxide, is produced on a commercial scale and costs approximately USD 150–250/kg (Made-in-China, 2024); market analysts estimate that the global market for ambroxide may reach USD 120 million by 2033 (Figure 1E) (Verified Market Reports, 2024).

This review summarises current knowledge on the distribution, physiological roles, identification, and analytical methods related to cis-abienol and its derivatives. It also discusses the biosynthetic and degradation pathways of cis-abienol and explores future directions for supporting the sustainable production of ambergris analogues.

## 2 Distribution and function of cis-abienol in tobacco leaves

In *N. tabacum*, glandular trichomes on the leaf surface excrete a rich array of diterpenoids, among which *cis*-abienol is the predominant labTD. The diterpenes comprise 10% of the dry weight of trichome secretions (Wang and Wagner, 2003). Cis-abienol is most abundant among the 20 labTD identified to date from the trichome exudates of tobacco (Caniard et al., 2012). It is produced in many *Nicotiana* species, particularly oriental and cigar tobacco, but its content can vary significantly between different genotypes and tissue types (Shi et al., 2024; Zhang et al., 2023). In one study, Ding Li et al. reported that the extraction of 0.845 mg/g cis-abienol from fresh oriental tobacco leaves (Ding et al., 2007).

Generally, it is believed that most standard flue-cured and burley tobaccos do not produce cis-abienol; however, researchers such as Qi et al. reported the extraction of cis-abienol from flue-cured *N. tabacum* germplasm lines cv. 8306 (Qi et al., 2023). Cis-abienol production is not restricted to oriental and cigar tobaccos. Evidence indicates that certain aromatic flue-cured cultivars, such as *Gexin No. 3*, *Henan Tobacco No. 11*, and *Dabaijin 599*, also synthesise this metabolite (Chang et al., 2018; Li et al., 2017). The compound displays strong tissue specificity, accumulating most heavily in flowers, followed by branches, leaves, stems, midribs, and finally fruits, indicating a higher biosynthetic capacity in reproductive than in vegetative tissues (He et al., 2021; Xu et al., 2022a).

**FIGURE 2:**
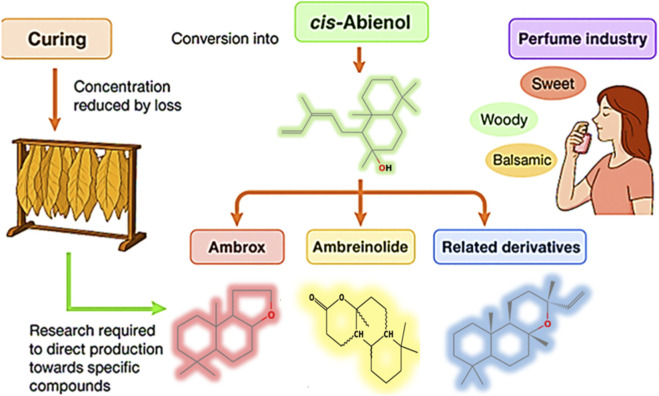
Role of cis-abienol in curing and its conversion to valuable aroma compounds for the perfume industry. The figure depicts how cis-abienol is transformed into Ambrox®, ambreinolide, and other derivatives during tobacco curing. Ambrox®, in particular, has attracted commercial interest as a sustainable substitute for ambergris in the perfume industry.

Cis-abienol is vital in shaping tobacco’s distinctive aroma profile, particularly in oriental tobaccos and certain cigar tobaccos, such as Havana cigar tobaccos, burley-derived aromatic wrapper tobaccos, Sumatran-type cigar tobaccos, and oriental–cigar hybrids (T. Cheng et al., 2020). The biosynthesis of cis-abienol in oriental tobacco is tightly associated with glandular trichome activity, which reaches its maximum during the later stages of leaf maturation. However, substantial losses of cis-abienol occur during subsequent air-curing and sun-drying, reflecting its instability under post-harvest processing conditions. The decline in cis-abienol during air-curing is attributed to oxidative degradation, volatilisation, and possible microbial activity (Shi et al., 2024) (Figure 2). Fu Qiujuan et al. demonstrated a ∼64% reduction in cis-abienol in the aromatic flue-cured cultivar Dabaijin 599, consistent with earlier reports showing that cis-abienol is highly susceptible to oxidation and volatilisation (Arnarp et al., 1993a; Arnarp et al., 1993b; Enzell and Wahlberg, 1990; Fu, 2020). These observations collectively indicate that cis-abienol is inherently unstable under curing conditions (Chang et al., 2018; Liu, 2023).

Air-curing also generates a diverse array of labdanoid derivatives: more than 20 LabTDs and over 25 degradation products of cis-abienol have been identified in oriental tobacco leaves, including Ambrox® and ambreinolide (Arnarp et al., 1993a; Arnarp et al., 1993b; Enzell and Wahlberg, 1990). Although Ambrox® may arise either through chemical synthesis or curing-induced transformation, the latter occurs under uncontrolled environmental conditions, resulting in less specific and lower-yielding oxidative cyclisation and molecular rearrangements compared with laboratory pathways (Koyama et al., 1987) (Figure 2).

**FIGURE 3:**
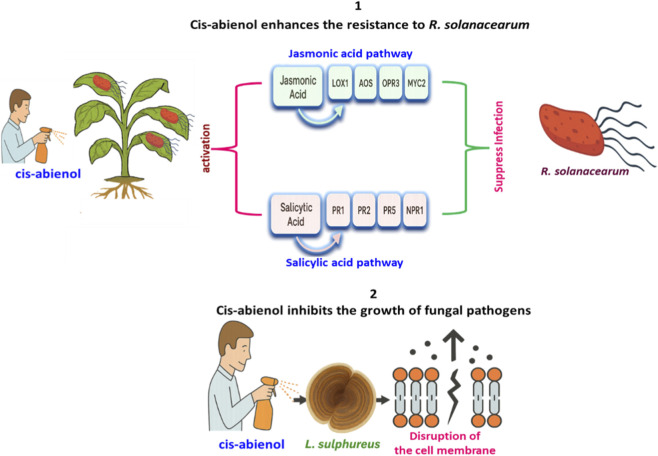
Broad-spectrum antimicrobial and defence-enhancing roles of cis-abienol in plants. This figure illustrates cis-abienol’s dual protective functions: (1) enhancing tobacco and tomato resistance to *Ralstonia solanacearum* by activating jasmonic acid (JA) and salicylic acid (SA) pathways, upregulating genes such as *LOX1*, *AOS*, *OPR3*, *MYC2*, *PR1*, *PR2*, *PR5*, and *NPR1*; and (2) inhibiting fungal pathogens like *Lenzites betulina*, *Laetiporus sulphureus*, *Gloeophyllum trabeum*, and *Trametes versicolor* through disruption of membrane integrity and interference with ergosterol biosynthesis. JA-Pathway Genes. LOX1 – Lipoxygenase 1, AOS–Allene oxide synthase, OPR3 – 12-oxophytodienoate reductase 3, MYC2 – Master JA-responsive transcription factor. Salicylic Acid (SA) Pathway Genes; These are classical SA-responsive defense genes, mainly induced against biotrophic pathogens: PR1 – Pathogenesis-related protein 1, PR2 – Pathogenesis-related protein 2 (β-1,3-glucanase), PR5 – Pathogenesis-related protein 5 (thaumatin-like protein), NPR1 – Nonexpressor of PR genes 1 (central SA signaling regulator).

Beyond its role in fragrance biosynthesis, cis-abienol also contributes to the plant’s innate defence mechanisms. In tobacco and other crop species, it has been shown to reduce susceptibility to insect herbivory, fungal pathogens, and bacterial infections (Wahlberg et al., 1977). Cis-abienol has demonstrated broad-spectrum biological activity, functioning both as an antimicrobial compound and as an inducer of systemic resistance in plants (Figure 3). For example, Sun et al. (2023) reported that exogenous application of cis-abienol at concentrations ranging from 60 to 80 μg/mL significantly enhanced resistance in tomato plants against *Ralstonia solanacearum* (the causal agent of bacterial wilt), without adversely affecting plant growth, photosynthetic efficiency, or biomass accumulation (Figure 3). The resistance response was associated with the upregulation of key genes in the jasmonic acid (JA) signaling pathway, including *LOX1*, *AOS*, *OPR3*, and *MYC2*, which exhibited transcript levels approximately 2.0–3.5 times higher than those in the control group. Similarly, in the salicylic acid (SA) pathway, the expression of *PR1*, *PR2*, and *PR5* was elevated by 2.5–4.0 fold, while *NPR1* expression increased by approximately threefold (Sun et al., 2023) (Figure 3). Consistent with these findings, Steede et al. (2017) reported that both foliar and root applications of cis-abienol in tobacco significantly reduced the severity of bacterial wilt, without compromising plant vigour. These results further support the role of cis-abienol as a resistance-inducing diterpenoid (Steede et al., 2017).

Cis-abienol extracted from *Cunninghamia lanceolata* (Chinese fir) exhibited potent antifungal activity against wood-decaying and plant-pathogenic fungi, including *Lenzites betulina, Laetiporus sulphureus, Gloeophyllum trabeum*, and *Trametes versicolor* (Cheng et al., 2012)(Figure 3). The cis-abienol inhibited the growth of fungal strains, possibly by disrupting their cell membranes through interference with sterol biosynthesis. (Cheng et al., 2012).

## 3 Analytical challenges and advances in cis-abienol detection

The detection and quantification of cis-abienol extracted from plant sources pose significant analytical challenges due to its poor thermal stability and susceptibility to degradation under high-temperature conditions (Liu et al., 2019). Although gas chromatography–mass spectrometry (GC–MS) is commonly employed for qualitative detection, the thermal lability of cis-abienol and its propensity to co-elute with structurally related diterpenes often result in suboptimal peak resolution (Liu et al., 2019).

GC–MS often fails to accurately quantify cis-abienol due to poor peak resolution and co-elution with structurally similar labdanoid diterpenes. Chang Aixia et al. reported unresolved cis-abienol peaks in volatile extracts of Samsun and Dabaijin 599 (Chang et al., 2018). Moreover, Wang Guoping et al. similarly observed incomplete separation in several high-producing genotypes, including Dabaijin 599, Gexin No. 3, NC2326, and TBT6 (Wang et al., 2020).

In contrast, liquid chromatography-based methods offer superior analytical performance. Fu Qiujuan et al. established a rapid and efficient UHPLC–UV method with low RSD values (2.05%–3.69%) and high separation efficiency (Fu, 2020). Sun Yuqing et al. achieved a purity of 96.24% using medium-pressure preparative chromatography followed by UHPLC purification. LC–MS approaches further enhance sensitivity and robustness; Liu Huaying et al. demonstrated that APCI–LC–MS provides fivefold improved detection sensitivity over ESI–LC–MS and prevents column fouling (Liu et al., 2019).

Collectively, these advances suggest that while GC–MS remains useful for the qualitative profiling of volatile diterpenes, UHPLC, HPLC, and APCI–LC–MS are preferable for the accurate and reproducible quantification of cis-abienol. The choice of method should consider not only detection limits and reproducibility but also compound stability, sample throughput, and the intended application.

## 4 Synthesis of cis-abienol

Ambergris, used in the perfume industry as a fixative to achieve a long-lasting fragrance, is nevertheless an expensive and scarce material. It is estimated that only 1% of sperm whales are capable of producing ambergris; therefore, the natural production of ambergris is extremely limited. Furthermore, sperm whales are classified as an endangered species, and the sale of ambergris in the United States and Australia is considered illegal (https://enviroliteracy.org/is-ambergris-banned-in-the-us/?utm_source=chatgpt.com). To balance the need to protect sperm whales with the growing demand for ambergris applications, synthetic ambergris compounds are increasingly favoured in the fragrance industry.

Cis-abienol, a natural LabTd, is a key precursor for synthesising high-value fragrance components such as ambroxide, a substitute for natural ambergris. Naturally, cis-abienol is found only in a few plant species, primarily in *Nicotiana tabacum* (tobacco), *Abies balsamea* (balsam fir), and *Calocedrus decurrens* (incense cedar) (Martínez-Guido et al., 2016). However, in these plants, it typically exists in trace amounts and is often part of complex resinous mixtures. Environmental factors, including soil composition, temperature, humidity, and light intensity, as well as seasonal and geographic variations, significantly impact the yield of cis-abienol in these plants. The accumulation of diterpene-like cis-abienol increased in balsam fir at the higher altitude and cooler climate (Allison and Paine, 2023). However, extracting cis-abeinol from plants is time-consuming, labour-intensive, and costly, as shown by its production cost of over $2,500 per kilogram (Martínez-Guido et al., 2016). Therefore, the commercial-scale extraction of cis-abienol from natural sources remains challenging; consequently, its applications are largely confined to perfumery and research.

Nevertheless, advances in metabolic engineering and synthetic biology have opened up promising routes for biosynthetic production of cis-abienol. These include: engineering tobacco and *Saccharomyces cerevisiae* to express the *NtCPS2* and *NtABS* genes involved in cis-abienol biosynthesis (i) CRISPR-based pathway optimisation in *Nicotiana benthamiana* (Xu et al., 2020) (ii) use of modular terpene production platforms in *E. coli* and *Streptomyces* species (Martínez-Guido et al., 2016; Pan and Ng, 2022; Xu et al., 2022b).

**FIGURE 4:**
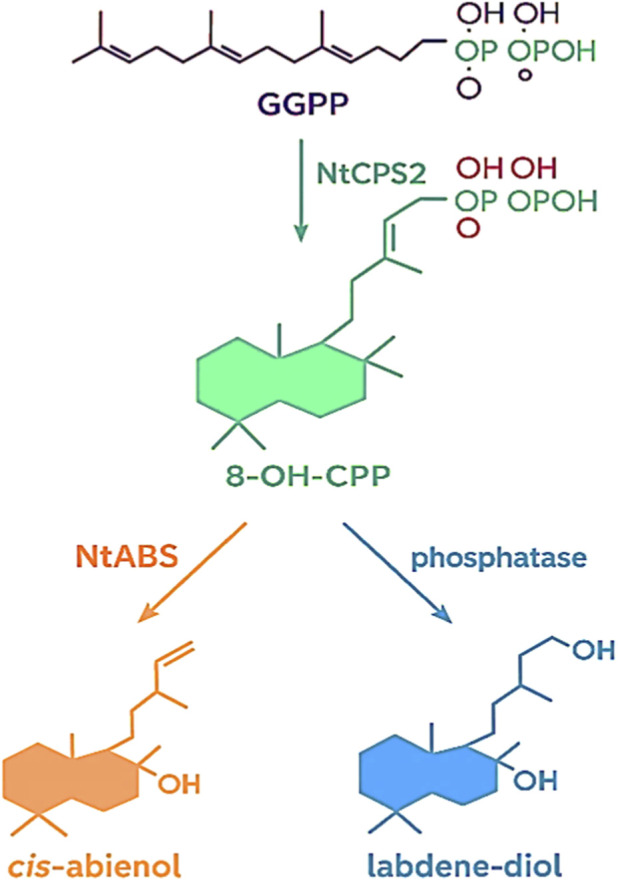
Biosynthesis pathway of biosynthesis of cis-abienol and labdene-diol. The enzyme NtCPS2 converts GGPP into 8-OH-CPP, which then branches into two possible routes. Through NtABS, it forms cis-abienol, while a phosphatase catalyzes the formation of labdene-diol.

Sallaud et al. successfully cloned two key genes, NtCPS2 (copalyl diphosphate synthase 2) and NtABS (abienol synthase), from aromatic tobacco *Basma Drama*, demonstrating the production of cis-abienol (Sallaud et al., 2012; Zerbe et al., 2012b). Specifically, NtCPS2 converts GGPP into 8-hydroxy-copalyl diphosphate (8-OH-CPP), which is subsequently used as a substrate by NtABS to produce cis-abienol (Figure 4). Functional genetic transformation in the diploid wild tobacco *Nicotiana sylvestris* further confirmed that both NtCPS2 and NtABS are required for cis-abienol biosynthesis (Zhang et al., 2023) (Figure 4). Additionally, Zerbe et al. also identified a cis-abienol synthase from *Abies balsamea,* which catalysed the conversion of GGPP into cis-abienol (Caniard et al., 2012; Zerbe et al., 2012a).

Zhang et al. genetically engineered *Escherichia coli* to enhance cis-abienol production by overexpressing genes from the mevalonate (MVA) pathway, along with ethanol kinase and isopentenyl phosphate kinase (IPK), thereby increasing the intracellular supply of dimethylallyl diphosphate (DMAPP). Furthermore, the authors deleted the *aphA* and *yqaB* genes, both of which encode aminoglycoside phosphotransferase A, leading to a significant increase in intracellular DMAPP accumulation. The engineered strain yielded 311.8 mg/L of cis-abienol after 112 h of fermentation in a 1.3 L bioreactor (Zhang et al., 2022). Similarly, Cheng et al. utilised *Escherichia coli* as a microbial chassis by introducing the mevalonate (MVA) pathway and co-expressing geranyl pyrophosphate synthase (GPPS), geranylgeranyl pyrophosphate synthase (GGPPS), and labda-13-en-8-ol synthase (LPPS). This metabolic engineering strategy enhanced precursor flux and resulted in the production of cis-abienol at a final titre of 220 mg/L (Cheng et al., 2020).

## 5 Transformation of cis-abienol and related labdanes

### 5.1 Chemical degradation and semisynthetic conversion of cis-abienol

**FIGURE 5:**
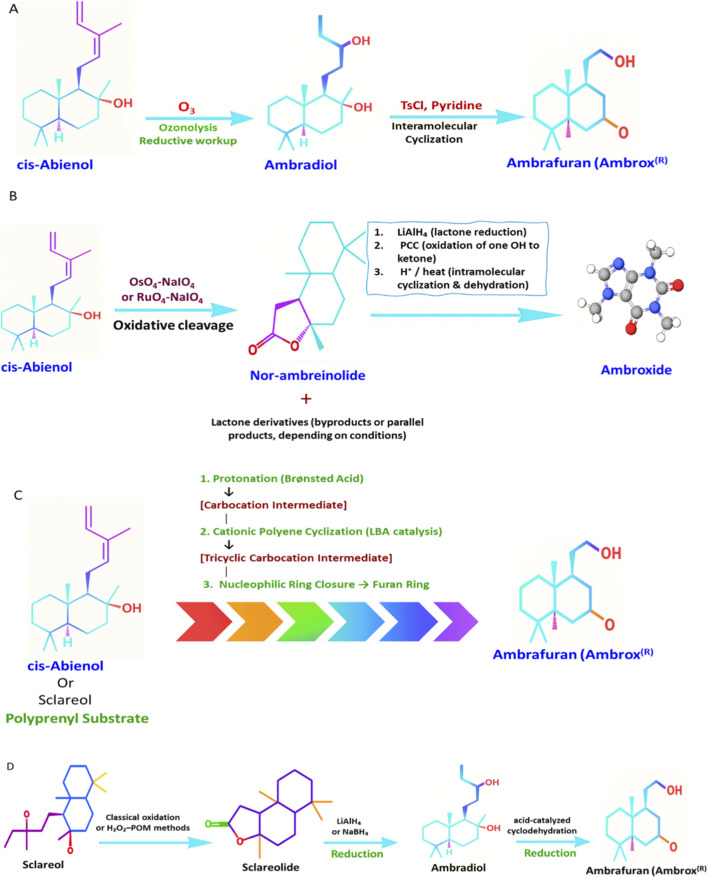
Overview of Classical, Oxidative, Cyclization, and Industrial Routes Leading from cis-Abienol to Ambrafuran and Ambroxide. **(A)** Illustrates the classical cis-abienol to ambrox® route; **(B)** presents the oxidative degradation pathway; **(C)** shows the stereoselective cyclization using the Ishihara–Yamamoto LBA system; **(D)** depicts the industrial parallel route derived from sclareol.

Chemical degradation of cis-abienol primarily targets its exocyclic diene side chain, enabling oxidative chain-shortening reactions that generate tetranorlabdane intermediates structurally close to Ambrox and other ambergris-like odorants. In one of the earliest and most influential studies, Barrero et al. demonstrated that ozonolysis of cis-abienol efficiently cleaves the C12–C13 diene bond, removing a four-carbon fragment and producing ambradiol, a stereochemically preserved tetranorlabdane diol. This intermediate serves as a direct chemical bridge between cis-abienol and the characteristic tricyclic skeleton of ambrafuran (Barrero et al., 1994a). Subsequent transformations, such as tosylation followed by intramolecular cyclodehydration, yield (−)-ambrafuran (Ambrox), representing one of the shortest reported semisynthetic routes to ambergris substitutes (Figure 5A).

Additional oxidative strategies have also been investigated. Ruthenium tetroxide and osmium tetroxide systems facilitate further oxidation of abienol-derived aldehydes to form lactone derivatives such as nor-ambreinolide, which retain the odor-active features associated with natural ambergris compounds (Figure 5B). These transformations illustrate that targeted oxidative degradation of cis-abienol can yield high-value fragrance intermediates through relatively short synthetic pathways (Barrero et al., 1993; Barrero et al., 1994a; Barrero et al., 1994b).

In addition to classical degradation-based approaches, advances in modern synthetic chemistry have enabled the *de novo* construction of the Ambrox framework through highly stereoselective cyclisation strategies (Figure 5C). A landmark study by Yamamoto and Ishihara Hideaki, (2002) introduced a Lewis-acid-assisted Brønsted acid (LBA) catalytic system that enabled highly controlled enantio- and diastereoselective cyclisation of polyprenyl substrates into complex terpenoid frameworks, including (−)-Ambrox. This mechanistically advanced cationic cascade illustrates that Ambrox can be synthesized not only via the degradation of natural labdane-type precursors, such as cis-abienol and sclareol, but also through the *de novo* construction of the furanolide core, thereby broadening the synthetic scope of ambergris-like fragrance chemistry (Figure 5D) (Yamamoto and Ishihara Hideaki, 2002).

While cis-abienol offers a compact two-step route to Ambrox, industrial production traditionally relies on sclareol, another plant labdane diterpene. The classical sclareol to Ambrox pathway proceeds via (i) oxidation to sclareolide, (ii) reduction to ambradiol, and (iii) acid-catalyzed cyclodehydration to yield ambrafuran. Process intensifications, green oxidants, and one-pot systems, such as the phosphomolybdate/H_2_O_2_ method, have been developed to improve atom economy and minimize waste, offering complementary alternatives to cis-abienol-based strategies (Ncube et al., 2020; Yang et al., 2016).

Together, these routes demonstrate that cis-abienol is a structurally privileged and renewable precursor for high-value ambergris substitutes, applicable to both classical and modern chemical systems (Figures 5A–D).

### 5.2 Biodegradation of cis-abienol

No effective biodegradation methods have been found to convert cis-abienol directly into ambergris. In contrast, research on its analog, sclareol, has been conducted relatively extensively (Han et al., 2025; Moulines et al., 2001). Sclareol has a similar structure to cis-abienol and serves as a key intermediate, produced through oxidation of the branched double bond in cis-abienol (Barrero et al., 1993; Moulines et al., 2001). It is also important for the production of ambrox and ambreinolide.

### 5.3 Biodegradation of sclareol

For the biodegradation studies of sclareol, Wolf-Rainer Abraham demonstrated that when *Bacillus sphaericus* ATCC 13805 was added to the culture medium with sclareol as the substrate, a 72-h fermentation catalyzed the conversion of sclareol into 3β-hydroxyaromadendrene and 18α-hydroxyaromadendrene (Abraham, 1994). Furthermore, using malt extract (17 g L^-1^) and mycological peptone (3 g L^-1^) as the medium, fermentation of sclareol with selected fungi converted it to 3-α-/3-β-hydroxysclareol and 3-oxosclareol, compounds employed as tobacco smoke flavor enhancers (Mitchell et al., 1984). Additionally, Mohamad I. Farbood and his team selected two microbial strains, *Cryptococcus albidus* (ATCC 20918, ATCC 2019) and *Hyphozyma roseonigra* (ATCC 20624), as biocatalysts to convert sclareol into sclareolide and sclareol glycol. Building on this biotransformation, they further synthesized ambroxide through subsequent chemical modification (Farbood et al., 1970).

S.A. Kouzi and colleagues investigated the microbial metabolism of sclareol, screening *Bacillus cereus* UI-1477 and identifying seven metabolites through fermentation, including 3β-hydroxyaromadendrene, 2α-hydroxyaromadendrene, and 18-hydroxyaromadendrene (Kouzi and McChesney, 1991a). They also employed *Septomyxa affinis* ATCC 6737 for preparative-scale fermentation of sclareol and successfully characterized three metabolites—8α,13β-dihydroxybenzo-14-en-3-one, labdane-14-en-3β,8α,13β-triol, and labdane-14-en-2α,8α,13β-triol—using 2D-NMR spectroscopy and chemical reactions (Kouzi and McChesney, 1990; Kouzi and Mcchesney, 1991b).

Furthermore, Huang Tingting and her team studied the metabolic regulation mechanisms of labdane diterpenoid compounds, discovering that cis-abienol, sclareol, and labdenediol could be converted into important products such as ambrox, ambreinolide, and nor-ambreinolide through oxidation, reduction, and cyclization reactions (Huang et al., 2019).

### 5.4 Biodegradation of cis-abienol

**FIGURE 6:**
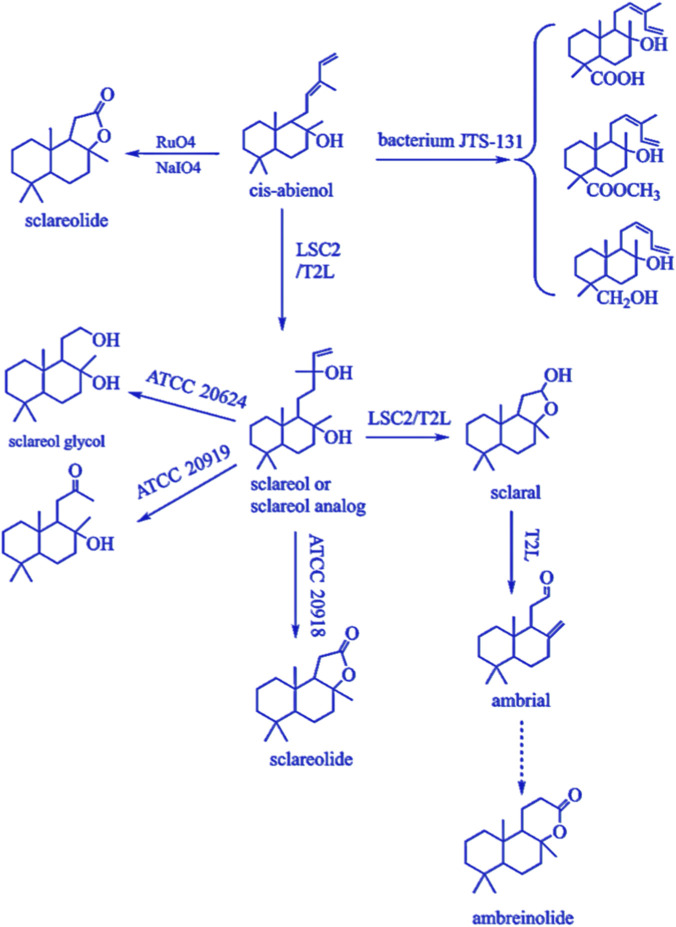
Biotransformation and oxidative pathways of cis-abienol and sclareol leading to ambergris derivatives: This figure illustrates the metabolic and chemical transformation pathways of cis-abienol and its analog sclareol.

Tadaharu Hieda et al. isolated a strain, JTS-131, capable of catalyzing the degradation of cis-abienol, which was identified as *Rhodococcus erythropolis* (Figure b). The major degradation products obtained from this strain included (12Z)-labdane-12,14-diene-18-ol, (12Z)-labdane-12,14-diene-18-carboxylic acid, and its methyl ester (Hieda et al., 1983). Further studies revealed that strain JTS-131 harbors a plasmid, pCA4, and its loss through curing treatment demonstrated that this plasmid is essential for the oxidation of the C-18 methyl group during cis-abienol fermentation (Hieda et al., 1983) (Figure 6).

*Acinetobacter tjernbergiae* (LSC-2), isolated from fresh oriental tobacco leaves and aromatic tobacco soil, degraded 69.3% of cis-abienol into metabolites such as amberonne isomers, sclareol analogs, and sclareol within 96 h (Figure 5) (Xi et al., 2025). Similarly, *Klebsiella oxytoca* sp. T2L was also isolated from fresh oriental tobacco leaves and soil, which degraded cis-abienol into products including sclaral, amberonne isomer analogs, and sclareol analogs (Figure 6). Remarkably, this strain significantly enhanced the yields of sclaral and amberonne isomers, with increases of 1,335% and 1,030%, respectively (Han et al., 2025) (Figure 6).

### 5.5 Sustainability and green chemistry aspects of cis-abienol bioconversion

The bioconversion of cis-abienol into Ambrox-like compounds (AmbLCs) offers a sustainable alternative to the use of natural ambergris and conventional multi-step petrochemical processes. Historically, ambrafuran and related ambergris odorants were obtained from ambergris, a rare intestinal excretion of the sperm whale, raising ethical and conservation concerns and prompting regulation of whale-derived materials. Subsequent industrial practice shifted to fully synthetic or semisynthetic ambrafuran made from petrochemical building blocks or plant terpenoids, including labdane diterpenes such as sclareol, labdanolic acid, and cis-abienol (Ncube et al., 2020).

In this context, cis-abienol offers several green-chemistry advantages. First, it is a renewable, plant-derived labdane diterpenoid that accumulates at high levels in tobacco glandular trichomes and in conifer oleoresins, and can now also be produced in engineered microbial cell factories from simple carbon sources such as glucose (Cheng et al., 2020). Metabolic engineering of *Escherichia coli* and yeast has achieved gram-scale cis-abienol production by reinforcing MVA/MEP pathway flux, demonstrating that cis-abienol can be generated from fermentable, biomass-derived feedstocks with high product specificity and without large-scale solvent extraction from plant biomass (Cheng et al., 2020). These developments align with the principles of green chemistry, which involve using renewable feedstocks and minimizing hazardous processing steps. Second, microbial biotransformation of tobacco-derived abienols into ambrox precursors further enhances sustainability.

Recent studies have identified *Acinetobacter tjernbergiae* LSC-2 as capable of converting Z-abienol into sclareol, scalaral, and amberonne, which are key intermediates in the biosynthesis of Ambrox and related ambergris-like odorants. This biotransformation pathway highlights a sustainable approach to bio-based fragrance production that utilises tobacco processing streams as feedstocks (Xi et al., 2025). Similarly, cis-abienol-degrading bacteria such as *Klebsiella oxytoca* T2L can generate ambreinolide, amberonne, and related ambergris-like compounds directly from cis-abienol, suggesting that dedicated biocatalysts could be integrated into tobacco-based biorefineries to valorise side-streams into high-value fragrance ingredients (Han et al., 2025).

Overall, these advances position cis-abienol bioconversion as a technically feasible and environmentally favourable route to AmbLCs. By combining renewable cis-abienol supply (from tobacco trichomes, conifers, or engineered microbes) with high-yield and biocatalytic conversions, the fragrance industry can reduce reliance on animal-derived ambergris and move toward processes that better satisfy multidimensional sustainability metrics (Ncube et al., 2020).

## 6 Conclusion

Cis-abienol is a critical intermediate for the industrial production of ambergris-type compounds. The biosynthesis of cis-abienol in the tobacco trichome has been extensively studied. The bioconversion studies of cis-abienol remain limited, and no highly efficient microbial pathways for its direct transformation into ambrox or ambreinolide have yet been established. The discovery of microbial strains such as *Rhodococcus erythropolis*, *Acinetobacter tjernbergiae*, and *Klebsiella oxytoca* capable of partially degrading cis-abienol highlights promising directions for further development. Advances in metabolic engineering, synthetic biology, and enzyme catalysis are expected to unlock sustainable biotechnological routes for the valorization of cis-abienol. Taken together, current developments in biochemical, metabolic engineering, and process chemistry indicate that cis-abienol is not only a key defence-related metabolite in tobacco but also a sustainable precursor for ambergris-like fragrance compounds. Plant- and microbe-derived cis-abienol can be produced from renewable feedstocks, and its conversion to ambrox or ambrafuran requires fewer synthetic steps, affording higher selectivity than many alternative routes. Emerging microbial biotransformations that upgrade tobacco-derived abienols into ambrox precursors further strengthen the case for cis-abienol-centered value chains, which add economic value to tobacco crops while reducing pressure on animal sources and enabling greener, more resource-efficient fragrance manufacture.
